# Fibulin-4 Accelerates Amyloid Formation by Binding with a Keratin 5 Peptide Fragment

**DOI:** 10.1016/j.xjidi.2022.100114

**Published:** 2022-03-09

**Authors:** Fumihiko Katagiri, Daisuke Ueo, Yumi Okubo-Gunge, Aya Usui, Sayaka Kuwatsuka, Yoshiko Mine, Keisuke Hamada, Sakuhei Fujiwara, Takako Sasaki, Motoyoshi Nomizu, Atsushi Utani

**Affiliations:** 1 Department of Clinical Biochemistry, School of Pharmacy, Tokyo University of Pharmacy and Life Sciences, Hachioji, Tokyo, Japan; 2 Department of Dermatology, Faculty of Medicine, Oita University, Yufu, Oita, Japan; 3 Department of Dermatology, Nagasaki University Graduate School of Biomedical Sciences, Nagasaki, Nagasaki, Japan; 4 Department of Biochemistry II, Faculty of Medicine, Oita University, Yufu, Oita, Japan

**Keywords:** CD, circular dichroism, K5, keratin 5, K5/14, keratin 5/14, PLCA, primary localized cutaneous amyloidosis

## Abstract

Keratins are the major amyloid fibril component in localized cutaneous amyloidosis. We analyzed the amyloid components in the skin of patients with localized cutaneous amyloidosis by immunohistochemical staining using antisera against extracellular matrix proteins and keratin 5 (K5). Fibulin-4 and K5 colocalized in the amyloid deposits. Using 14 synthetic peptides, we screened for amyloidogenic sequences in the C-terminal region of K5, including the α-helical rod domain and the tail domain. Two peptides stained with thioflavin T possessed a β-sheet structure and formed amyloid-like fibrils. Among the amyloidogenic peptides, a peptide KT5-6 (YQELMNTKLALDVEIATYRKLLEGE) derived from the α-helical rod domain of K5 specifically bound to fibulin-4. In addition, amyloid formation of KT5-6 was accelerated by fibulin-4. These results suggest that degraded fragments of K5 containing the KT5-6 sequence form amyloid fibrils with fibulin-4. The data further suggest that degraded fragments of K5 and fibulin-4 have the potential to initiate cutaneous amyloidosis.

## Introduction

Amyloidosis is caused by abnormal extracellular matrix deposition and accumulation of insoluble fibrous proteins that have a β-sheet structure. Amyloidosis can result in the dysfunction of either the whole body or specific organs (Glenner, 1980). Localized cutaneous amyloidosis has been divided into primary localized cutaneous amyloidosis (PLCA) and secondary localized cutaneous amyloidosis (Wong, 1987). Both types of localized cutaneous amyloidosis are associated with the deposition of amyloid only in the skin, without the involvement of internal organs. Lichen amyloidosis is the most common form of PLCA and ordinarily presents as relentless pruritic plaques on the extensor surfaces of the lower legs and on the forearms and back (Bolognia et al., 2008; Wang, 1990; Westermark et al., 1999). Secondary localized cutaneous amyloidosis is observed in skin tumors, including basal cell carcinomas, Bowen’s carcinoma, and benign skin tumors (Breathnach, 1988; Breathnach and Hintner, 1990).

The pathogenesis of localized cutaneous amyloidosis is not fully understood, although immunohistochemical studies suggested that cutaneous amyloid deposits are mainly composed of keratins (Kobayashi and Hashimoto, 1983; Maeda et al., 1982; Masu et al., 1981). Previously, galectin-7 and actin, in addition to keratins, were found in some cases of both PLCA and secondary localized cutaneous amyloidosis (Miura et al., 2013). Galectin-7, actin, and keratins are the major constituents of PLCA. The tryptic peptides of galectin-7 released at neutral pH may lead to PLCA amyloid fibril formation in the acidified intracellular environment during keratinocyte apoptosis through the interaction of the galectin-7 peptides with both actin and keratins (Ono et al., 2014). Previous immunohistochemical and electron microscopic studies revealed that elastic fibers were present in the center of fibrous amyloid deposits in some cases of cutaneous amyloidosis (Yanagihara et al., 1990, 1985). These data suggest that component(s) of the extracellular matrix might be the scaffold for cutaneous amyloid; however, the precise molecular function of the elastic component(s) is unknown. Only a few biochemical studies of cutaneous amyloid have been performed (Ono et al., 2014) because the amyloid components are hard to purify owing to their low amounts.

Keratins are heterodimeric proteins composed of a central α-helical coiled‒coil rod domain and nonhelical head and tail domains. The coiled‒coil domain is highly conserved in the keratin family (Lee et al., 2012). Keratin 5/14 (K5/14) mainly locates in the basal cell layers, and keratin 1/10 mainly locates in the suprabasal cell layers (Chang et al., 2004). K5/14 is the major amyloid fibril component on the basis of immunohistochemical studies, but detailed biochemical analyses have not been performed.

Elastic fibers provide elasticity and resilience to the skin. The major component of elastic fibers is elastin, and it assembles into a complex fibrous structure together with a number of microfibrillar proteins. Fibulin-4 is one of the elastin-associated proteins and is especially indispensable for elastic fiber formation (McLaughlin et al., 2006). Mutations in the human fibulin-4 gene, *EFEMP2*, also known as *FBLN4*, cause autosomal recessive cutis laxa type IB characterized by severe systemic connective tissue abnormalities (Uitto et al., 2013).

In this study, we focused on keratin 5 (K5) and the extracellular matrix proteins, which are components of elastic fiber, and performed immunohistochemical staining of lesional skin sections of Bowen’s carcinoma, lichen amyloidosis, and familial PLCA using antibodies against elastic fiber proteins and K5. The data showed the accumulation of K5 and fibulin-4 in cutaneous amyloid deposits. We identified an amyloidogenic sequence in K5 using a set of synthetic peptides. Furthermore, the effect of fibulin-4 on promoting the amyloid formation of a keratin peptide was also shown.

## Results

### K5 and fibulin-4 were found in amyloid deposits

**Figure 1:**
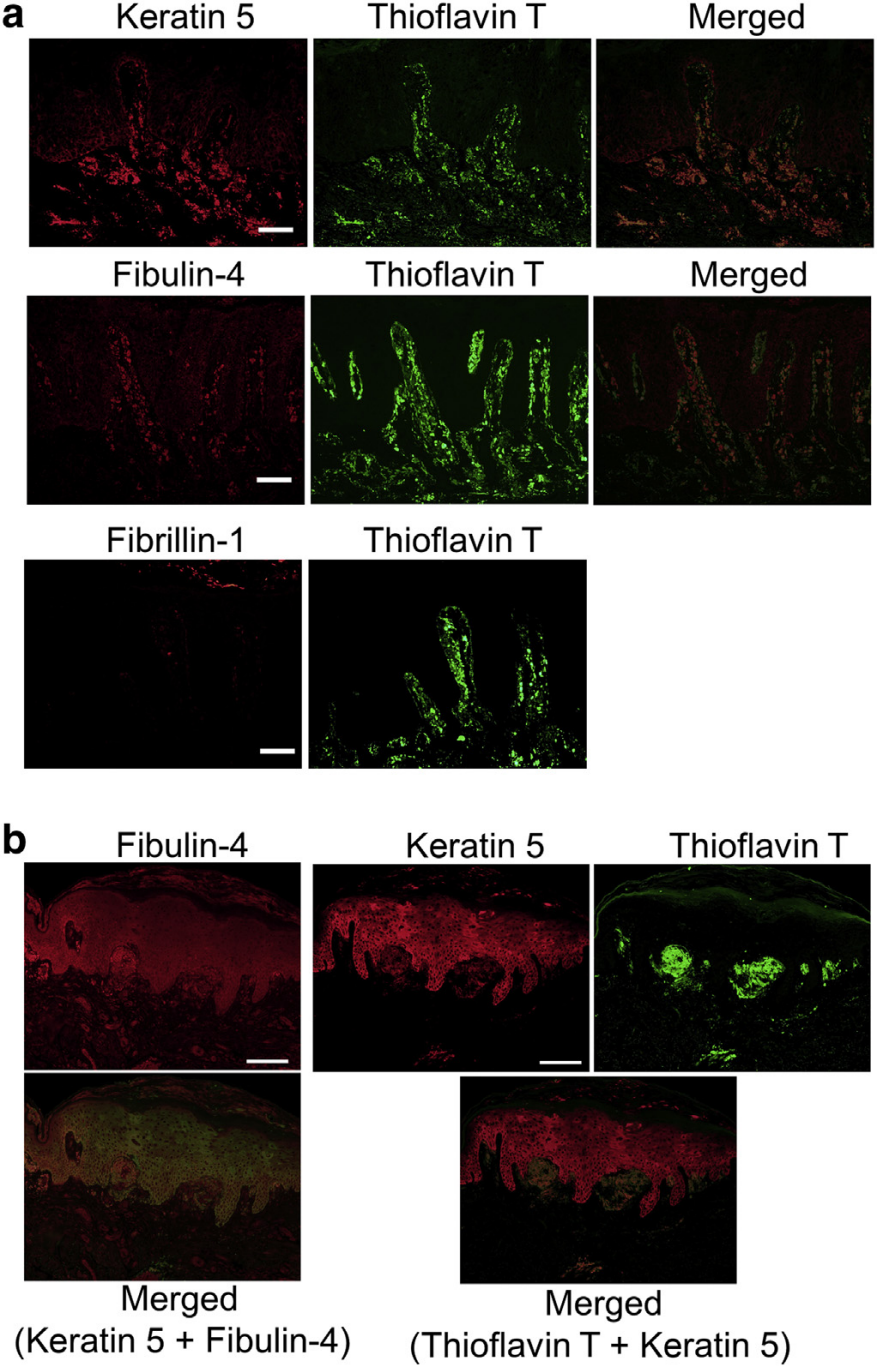
**Keratin 5 and fibulin-4 were found in the amyloid deposits of Bowen’s carcinoma and lichen amyloidosis skin.** (a) The skin from a Bowen’s carcinoma (patient 1) was stained with anti‒fibulin-4, anti‒keratin 5, and anti‒fibrillin-1 antibodies (the left column) together with thioflavin T. Bar = 50 μm. (**b**) Paraffin-embedded sections (patient 2, lichen amyloidosis) were stained with anti‒fibulin-4 (red) and anti‒keratin 5 (green, data not shown) and with anti‒keratin 5 and thioflavin T. Bar = 100 μm.

**Figure 2:**
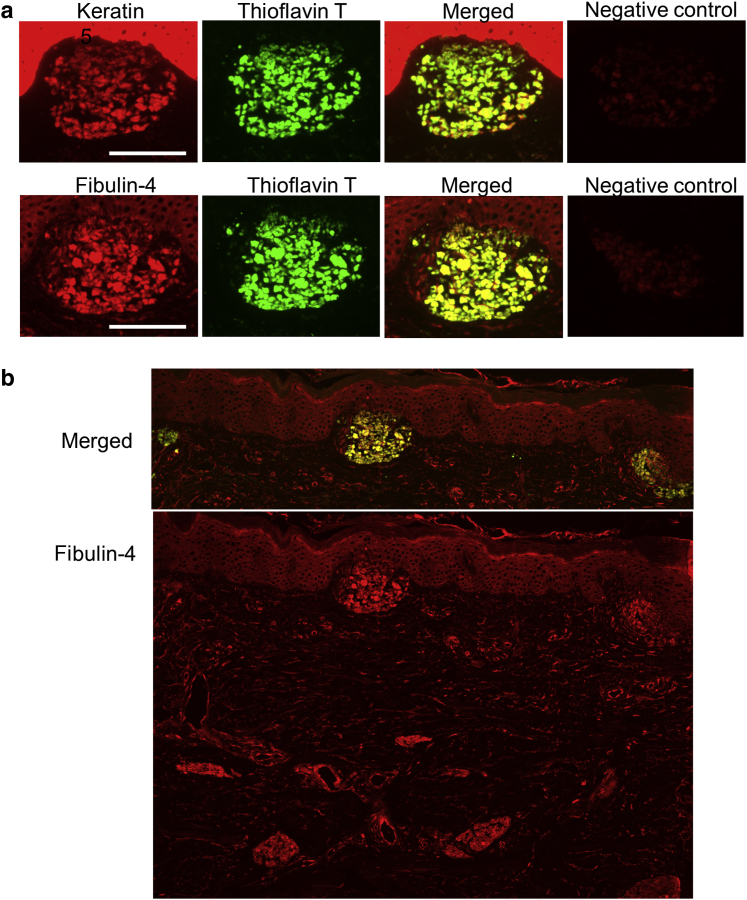
**Fibulin-4 and keratin 5 were found in the amyloid deposits of lichen amyloidosis (patient 3).** (**a**) Consecutive frozen sections were stained with anti-keratin 5 or with anti‒fibulin-4, and amyloid deposits were detected with thioflavin T. Each merged image is also shown. (**b**) The staining with anti‒fibulin-4 in the same section as the second row of **a** was shown at lower magnification. This image also shows the localization of fibulin-4 in normal human skin. Fibulin-4 localizes in elastic fibers and in the surrounding blood vessels, not only in the papil1ary dermis but also in the reticular dermis. Bar = 100 μm.

**Figure 3:**
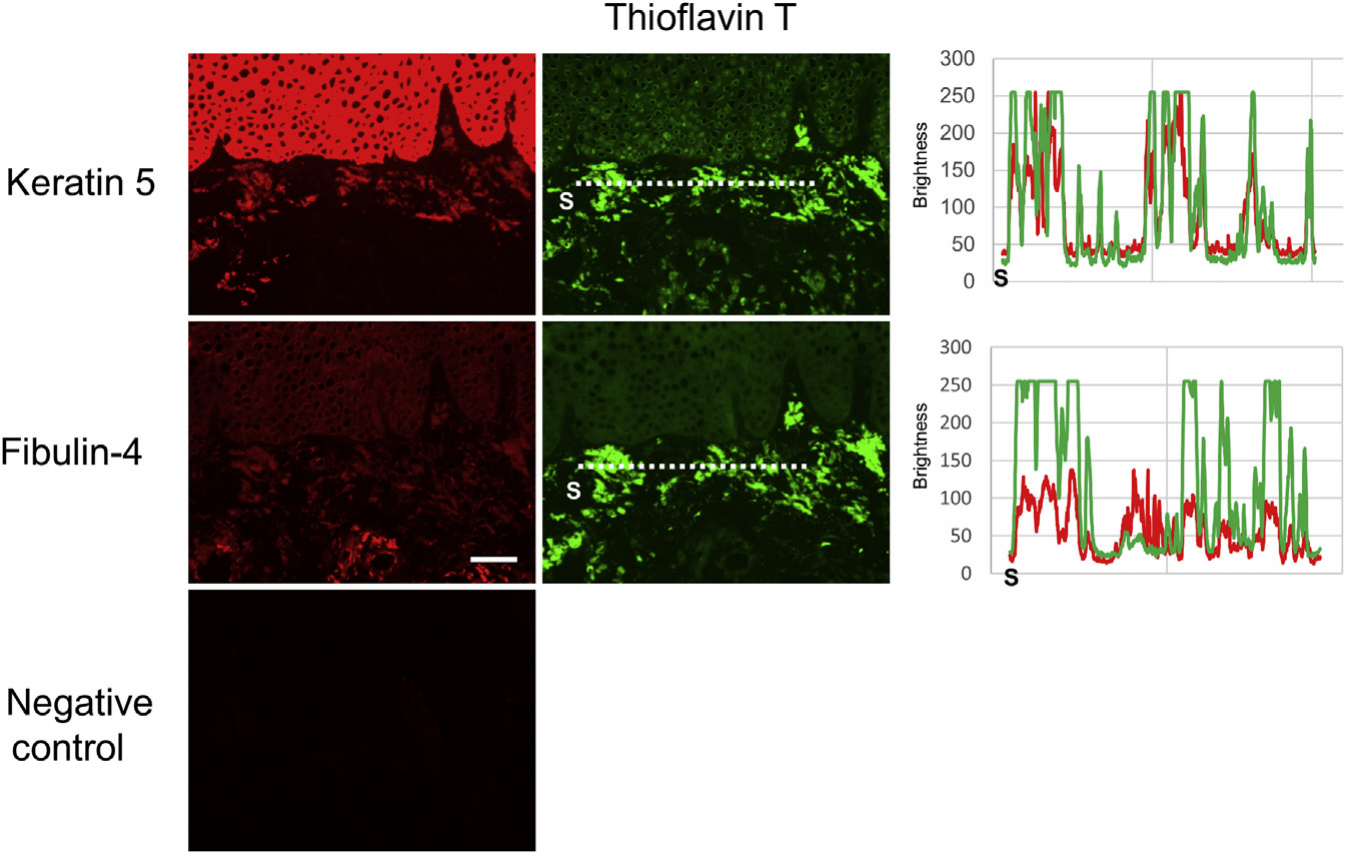
**The amyloid deposits in lichen amyloidosis skin derived from patient 4.** Keratin 5 and fibulin-4 colocalized in the amyloid deposits detected with thioflavin T; however, the staining with anti‒fibulin-4 was very weak. Bar = 50 μm. The fluorescence intensity along the dotted line was shown at the right panel showing the colocalization of thioflavin T and keratin 5/fibulin-4. S indicates the starting point of the measurement.

**Figure 4:**
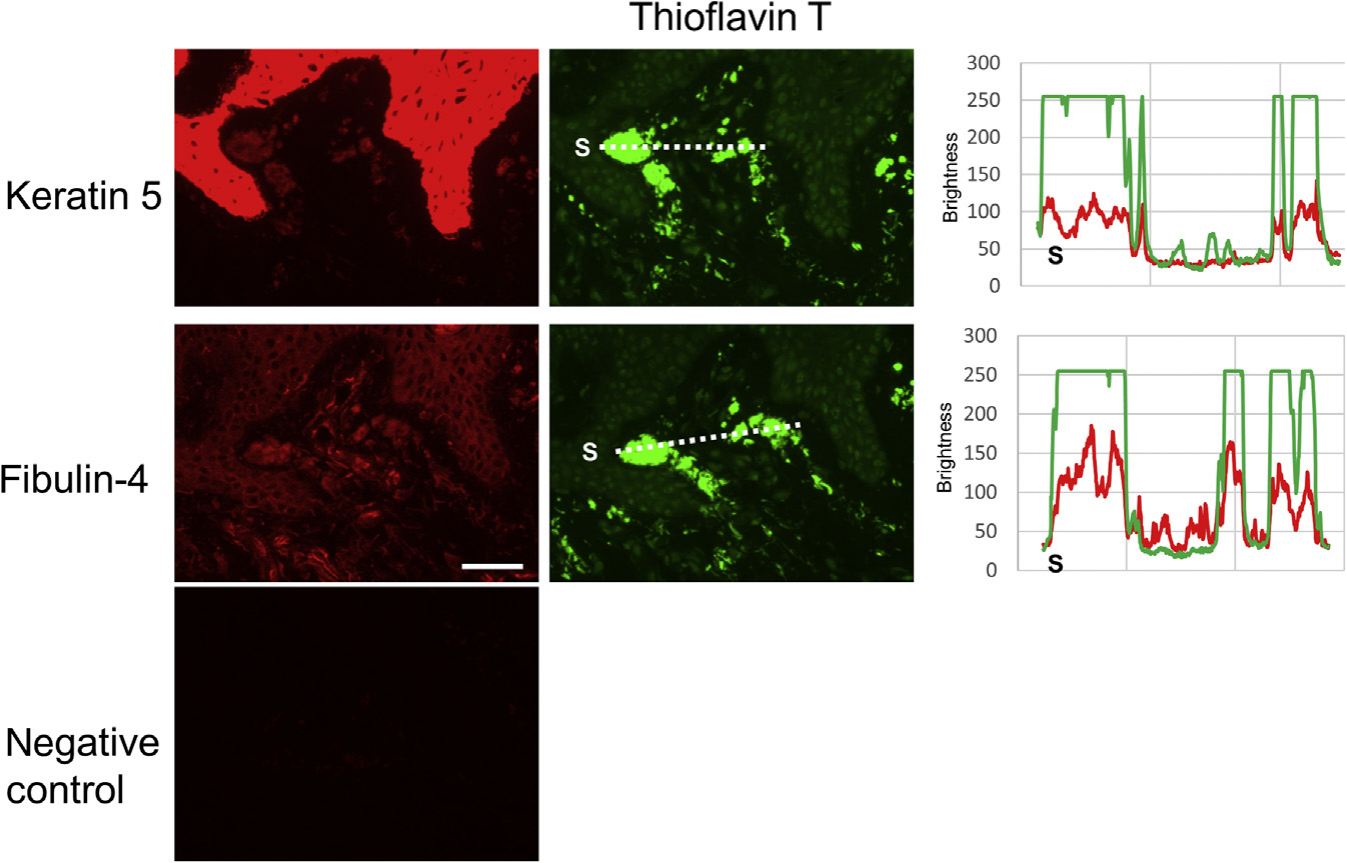
**The amyloid deposits of familial PLCA skin derived from patient 5.** Keratin 5 and fibulin-4 were found in the amyloid deposits. Bar = 50 μm. The fluorescence intensity along the dotted line was shown at the right panel showing the colocalization of thioflavin T and keratin 5/fibulin-4. S indicates the starting point of the measurement. PLCA, primary localized cutaneous amyloidosis.

**Figure 5:**
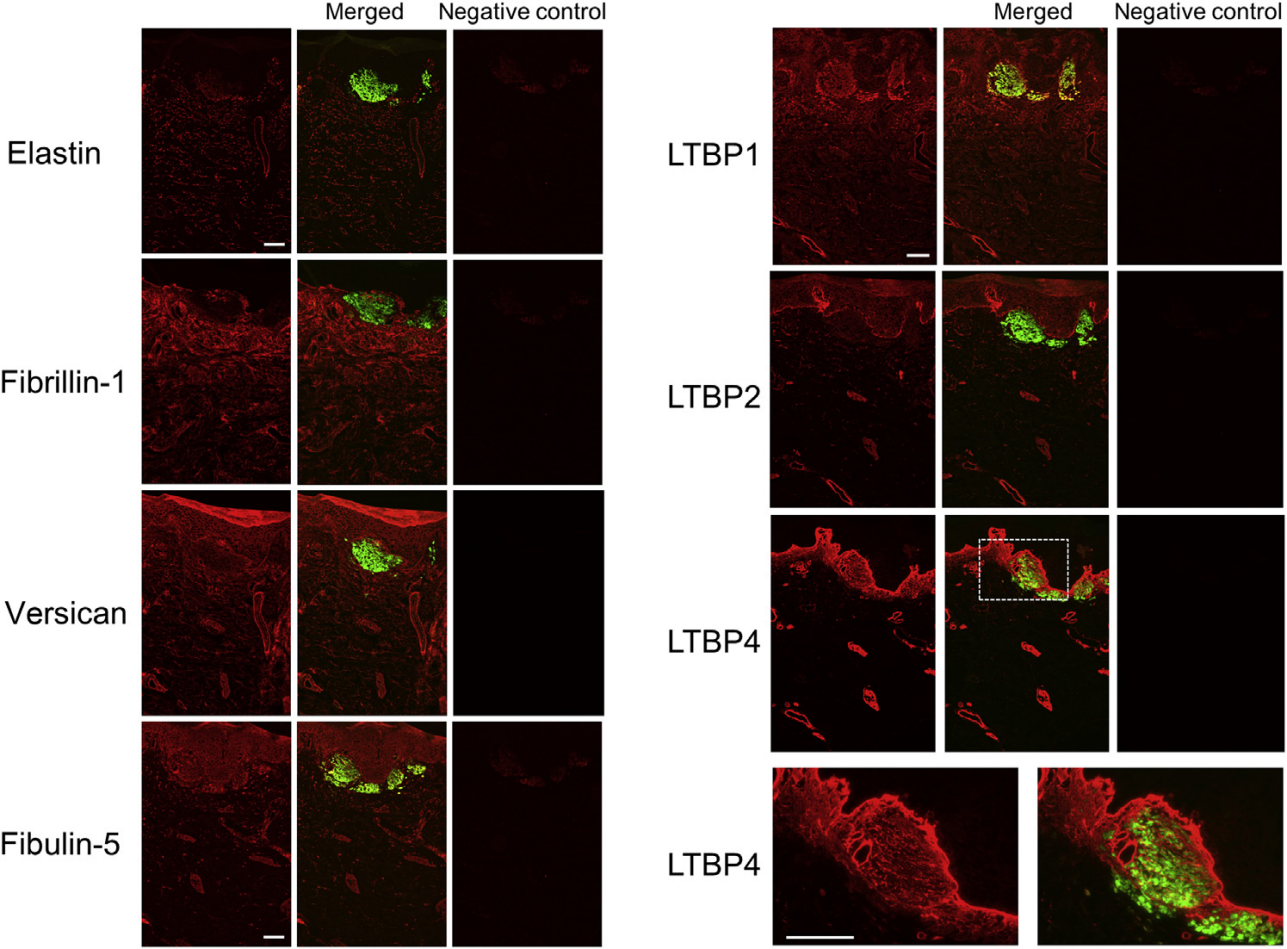
**Immunohistological analyses using the antibodies against elastic fiber components: elastin, fibrillin-1, versican, LTBP1, LTBP2, LTBP4, and fibulin-5.** Skin sections were derived from patient 3. The merged pictures with thioflavin T staining were shown at the middle panel, and each negative control was shown at the right panel. Bar = 100 μm. The staining with anti-LTBP4 was shown also at higher magnification (bar = 100 μm) to show that LTBP4 was not accumulated in amyloid. LTBP1 and fibulin-5 were abnormally deposited in the amyloid, but other proteins were not. Skins from patients 4 and 5 were also stained, and data were similar (data not shown).

Lesional skin sections were stained with antibodies against K5 and elastic fiber proteins. K5 and fibulin-4 were found in the amyloid deposits, which were visualized with thioflavin T (Sigma-Aldrich, St. Louis, MO), a stain specifically for amyloid structures, in the skin derived from Bowen’s carcinoma (patient 1, Figure 1a), lichen amyloidosis (patients 2‒4, Figures 1b, 2a, and 3) and familial PLCA due to a heterozygous missense mutation in *OSMRβ* (Ueo et al., 2016) (patient 5, Figure 4), although fibulin-4 staining was weaker in the skins from patient 4 (Figure 3) and patient 5 (Figure 4). In normal skin, the expression of K5 is restricted to the epidermis. In contrast, fibulin-4 can be found throughout the dermis (Figure 2b). In the amyloid of these skin samples, fibulin-4 accumulated and colocalized with K5. Abnormal localization in amyloid deposits was also detected for LTBP1 and fibulin-5 and weakly for LTBP2. However, other elastic fiber proteins, including elastin, fibrillin-1, versican, and LTBP4, did not accumulate in the amyloid deposits (Figure 1a, in the bottom row, and Figure 5). These data raised the possibility that the interaction between K5 and fibulin-4/LTBP1/fibulin-5 is involved in amyloid formation.

### Synthetic peptides covering the K5 C-terminal sequence

**Figure 6:**
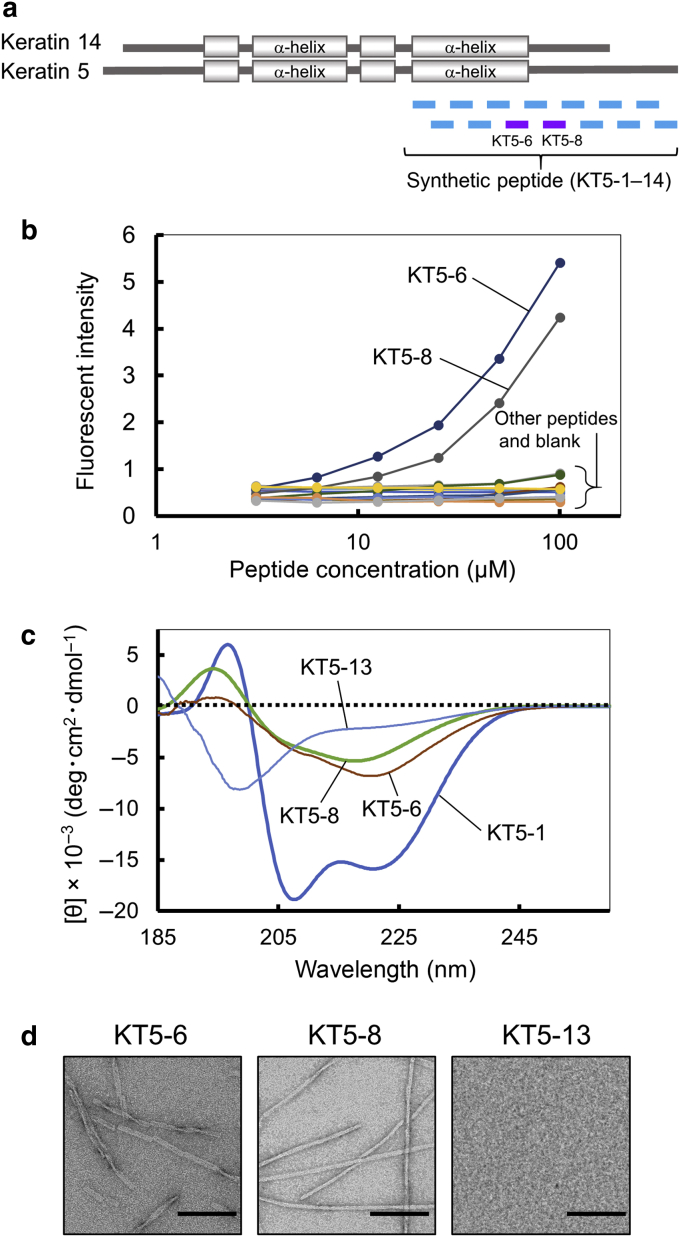
**Physicochemical properties of peptides derived from the C-terminal region of keratin 5.** (**a**) Localization of synthetic peptides in the keratin 5/14 molecule. (**b**) Dose-response curve for peptides measured with thioflavin T. Thioflavin T solution (50 μl, 20 μM in 0.2 mM glycine‒NaOH buffer [pH 8.5]) was added to the peptide solutions (50 μl, 3.125‒100 μM in H_2_O) in a 96-well plate, and the fluorescent intensity was measured immediately at an excitation wavelength of 455 nm and emission wavelength at 490 nm. (**c**) CD spectra of keratin peptides. (**d**) Electron micrograph of keratin peptide amyloid-like fibrils. Bar = 100 nm. CD, circular dichroism; H_2_O, water; NaOH, sodium hydroxide.

K5 is composed of a central α-helical rod domain and nonhelical head and tail domains. The central α-helical rod domain is highly conserved in the keratin family and plays a critical role in dimerization through a coiled‒coil assembly with a paired keratin molecule (Lee et al., 2012). K5 forms a heterodimer with keratin 14. We focused on the C-terminal region of K5, including the α-helical rod domain and the tail domain, as having a potential role in amyloid formation. A total of 14 synthetic peptides were prepared covering these sequences (Figure 6a and Table 1). Each peptide contained over 18 amino acid residues, overlapping four amino acid residues with the neighboring peptides. KT5-1‒KT5-6 are located in the α-helical rod domain, and KT5-7‒KT5-14 are located in the tail domain.

**Table 1:** List of Keratin Peptides and Their Activities

Peptide	Sequence (Position^1^)	Thioflavin T^2^	CD^3^
KT5-1	TKHEISEMNRMIQRLRAEI (382‒400)	‒	α-helix
KT5-2	RAEIDNVKKQCANLQNAI (397‒414)	‒	α-helix
KT5-3	QNAIADAEQRGELALKDA (411‒428)	‒	random
KT5-4	LKDARNKLAELEEALQKA (425‒442)	‒	α-helix
KT5-5	LQKAKQDMARLLREYQEL (438‒456)	‒	α-helix
KT5-6	YQELMNTKLALDVEIATYRKLLEGE (453‒477)	+	β-sheet
KT5-7	EGEECRLSGEGVGPVNIS (475‒492)	‒	random
KT5-8	VNISVVTSSVSSGYGSGS (489‒506)	+	β-sheet
KT5-9	GSGSGYGGGLGGGLGGGL (503‒520)	‒	random
KT5-10	GGGLGGGLAGGSSGSYYS (517‒534)	‒	random
KT5-11	SYYSSSSGGVGLGGGLSV (531‒548)	‒	random
KT5-12	GLSVGGSGFSASSGRGLG (545‒562)	‒	random
KT5-13	RGLGVGFGSGGGSSSSVK (559‒576)	‒	random
KT5-14	SSVKFVSTTSSSRKSFKS (573‒590)	‒	random
KT1-6	YQELMNTKLALDLEIATYRTLLEGE	+	β-sheet
KT14-6	YKILLDVKTRLEQEIATYRRLLEGE	‒	α-helix

Abbreviation: CD, circular dichroism.

1 Position of the peptides in the human keratin 5 sequence was described.

2 Effect of the fluorescent intensity in the thioflavin T analysis was described.

3 Secondary structure of the peptides in the CD spectroscopy analysis was shown.

### Thioflavin T staining and circular dichroism spectroscopy analysis of synthetic peptides

First, we examined amyloid formation by synthetic peptides using thioflavin T (Figure 6b). Peptides KT5-6 and KT5-8 increased the fluorescent intensity of thioflavin T in a dose-dependent manner. In contrast, none of the other peptides affected the fluorescent intensity in the thioflavin T analysis. These results suggest that KT5-6 and KT5-8 promote amyloid formation.

We also analyzed the secondary structure of the peptides using circular dichroism (CD) spectroscopy. KT5-6 and KT5-8 possessed a typical β-sheet CD spectrum pattern (Figure 6c). In contrast, KT5-1, KT5-2, KT5-4, and KT5-5 showed a typical α-helix CD spectrum pattern (Table 1). A CD spectrum of KT5-1 is shown in Figure 6c. The other peptides, including KT5-13, showed a random coil structure in the CD analysis (Figure 6c and Table 1). These results indicate that KT5-6 and KT5-8 possess a β-sheet structure and have the potential to form an amyloid-like structure.

### Electron micrograph of synthetic peptides

KT5-6 and KT5-8, which showed amyloidogenic activity in the thioflavin T analyses and a β-sheet structure in the CD analyses, were examined using electron microscopy (Figure 6d). KT5-13 was also examined as controls. KT5-6 and KT5-8 formed amyloid-like fibrils. In contrast, KT5-13 did not form amyloid-like fibrils.

### Binding of keratin synthetic peptides to fibulin-4

**Figure 7:**
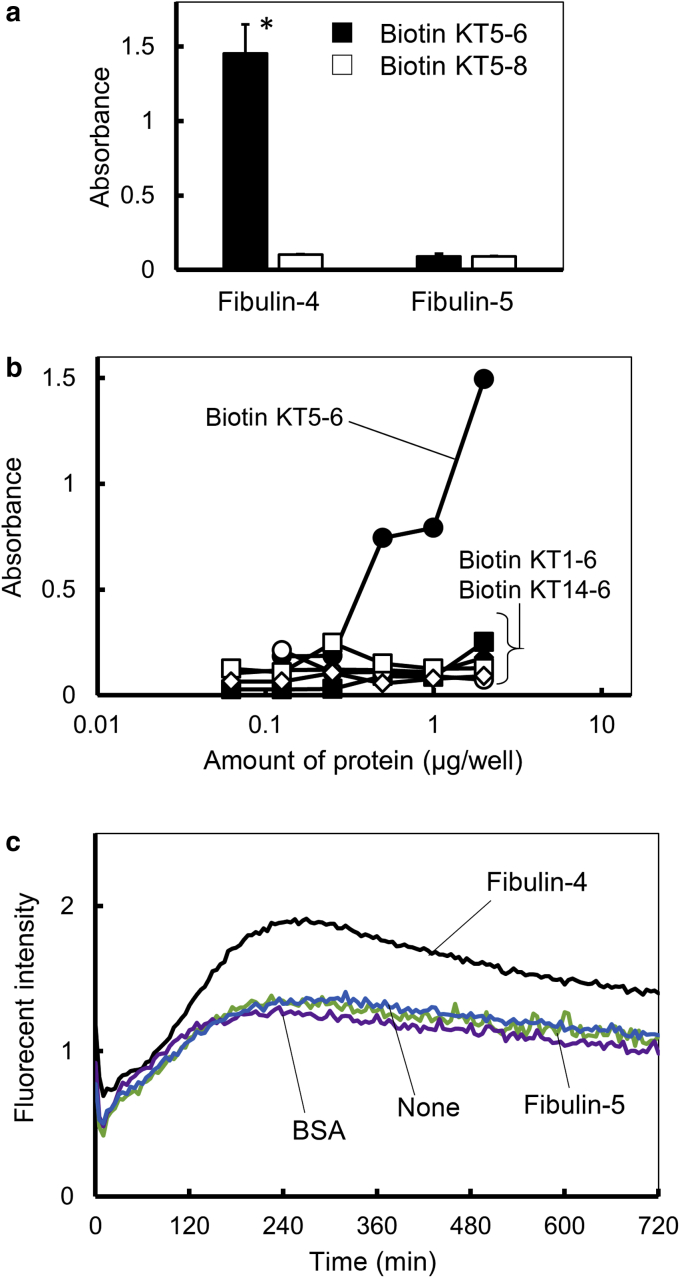
**Binding of keratin peptides to fibulins.** (**a**) Biotin KT5-6 binds to fibulin-4 but not to fibulin-5, whereas biotin KT5-8 does not bind to either fibulin. Results were expressed as means + SD. Comparison of mean values was performed using repeated measures one-way ANOVA and a Welch’s *t*-test. ∗*P* < 0.01, significantly different from biotin-KT5-8 on fibulin-4. (**b**) Biotin-KT5-6 binds to fibulin-4 in a dose-dependent manner but not fibulin-5, whereas none of the homologous KT5-6 peptides does bind to either fibulin. (**c**) Kinetic analysis of KT5-6 amyloid formation with fibulins. min, minute.

The binding of KT5-6 and KT5-8 to fibulin-4 was examined using biotinylated KT5-6 and KT5-8 (biotin KT5-6 and biotin KT5-8) peptides and fibulin-4‒coated plates. Fibulin-5‒coated plates were used as a control. Biotin KT5-6 and biotin KT5-8 were added to fibulin-4‒ and fibulin-5‒coated plates, and the binding of the peptides was analyzed using streptavidin horseradish peroxidase (Figure 7a). Biotin KT5-6 bound to the fibulin-4‒coated plates, but biotin KT5-8 did not bind to the fibulin-4‒coated plates (Figure 7a). In addition, both biotin peptides did not bind to the fibulin-5‒coated plates. These results suggest that KT5-6 specifically binds to fibulin-4.

### Amyloidogenicity and fibulin binding of KT5-6 homologous peptides

Because KT5-6 formed amyloid-like fibrils and bound to fibulin-4, we examined the amyloidogenicity of KT5-6 homologous peptides. K5 and keratin 14 assemble in the coiled‒coil domain and form a heterodimer K5/14. The KT5-6 sequence locates in the C-terminal end of the coiled‒coil domain, and the region is highly conserved in the keratin family (Lee et al., 2012). We synthesized a KT5-6 peptide homologous to keratin 14, KT14-6 (Table 1). Previous crystallization studies showed that the KT5-6 and KT14-6 sequences interact in the K5/14 molecule (Lee et al., 2012). K5/14 mainly locates in the basal cell layers, and keratin 1/10 mainly locates in a suprabasal cell layer of the epidermis. We also synthesized a KT5-6 homologous peptide of keratin 1, KT1-6 (Table 1). KT1-6 and KT14-6 were examined for their amyloidogenicity. KT1-6 increased the fluorescent intensity in the thioflavin T analysis, possessed a typical β-sheet CD spectrum pattern, and formed amyloid-like fibrils (data not shown, Table 1). In contrast, KT14-6 did not affect the fluorescent intensity in the thioflavin T analysis and possessed a typical α-helix CD spectrum pattern (data not shown, Table 1). These results suggest that KT1-6 has amyloidogenicity similar to that of KT5-6, but KT14-6 does not possess this activity.

The binding of KT5-6, KT1-6, and KT14-6 to fibulin-4 and fibulin-5 were evaluated using their respective biotinylated peptides. Biotin KT5-6, biotin KT1-6, and biotin KT14-6 were added to fibulin-4‒ and fibulin-5‒coated plates, and binding of the peptides was analyzed using streptavidin horseradish peroxidase (Figure 7b). Biotin KT5-6 bound to the fibulin-4‒coated plates in a dose-dependent manner, but biotin KT1-6 and biotin KT14-6 did not. Furthermore, none of the biotinylated peptides bound to the fibulin-5‒coated plates. These results suggest that KT5-6 specifically binds to fibulin-4.

### Effect of fibulin-4 on the amyloid formation of KT5-6

Next, the effect of fibulin-4 on KT5-6 amyloid formation was examined (Figure 7c). Biotin KT5-6 and thioflavin T were added to either fibulin-4‒, fibulin-5‒, or BSA-coated plates, and the fluorescent intensity was measured with time. When biotin KT5-6 and thioflavin T were added to the fibulin-4‒coated plates, the fluorescent intensity was significantly increased and reached a plateau in 240 minutes. When biotin KT5-6 and thioflavin T were added to the fibulin-5‒coated plates, the fluorescent intensity increased to a plateau in 240 minutes, but the intensity was weaker than that on the fibulin-4‒coated plate and was similar to that on the BSA-coated or uncoated plates. These results suggest that fibulin-4 has a role in promoting KT5-6 amyloid formation.

## Discussion

**Figure 8:**
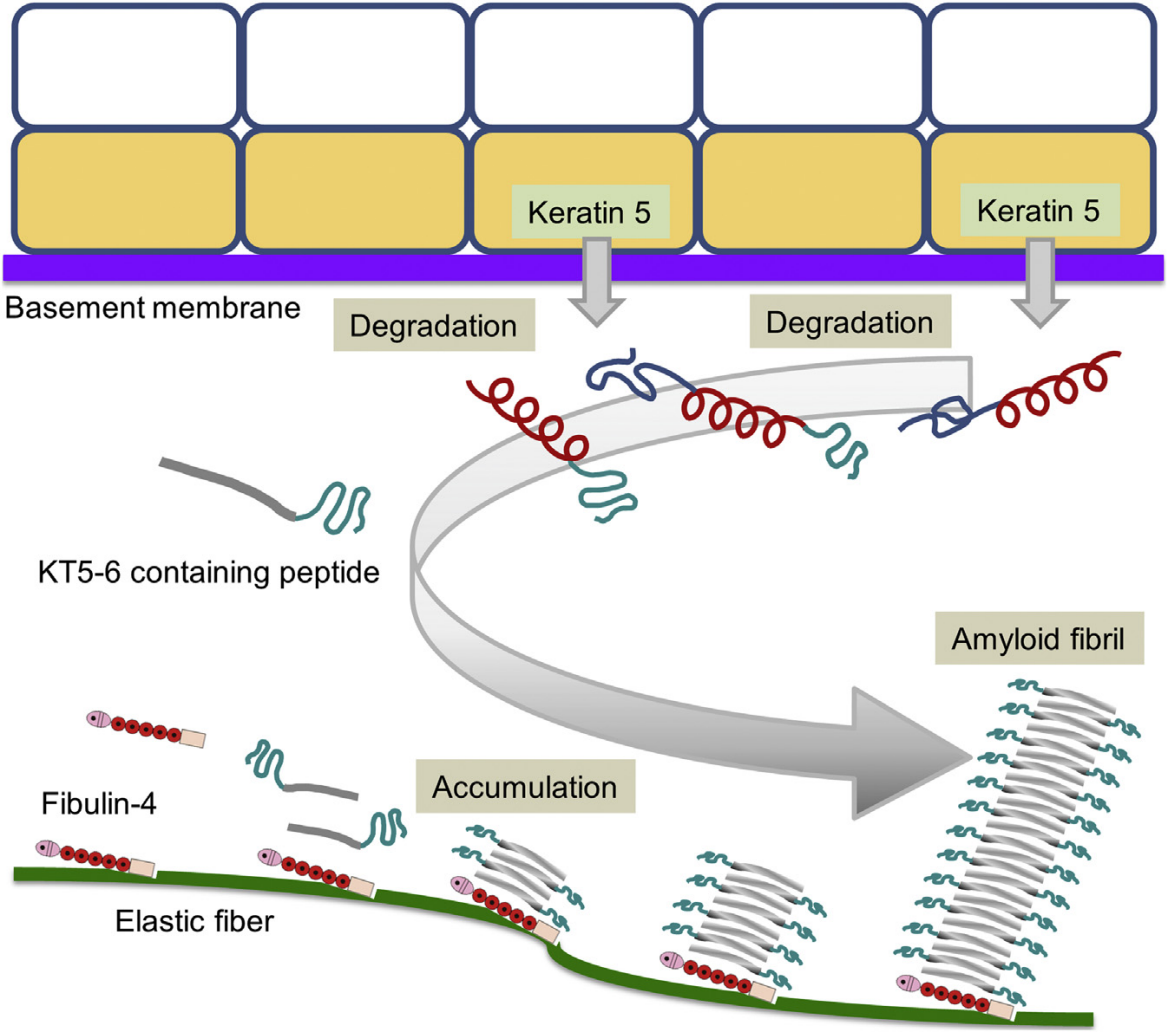
**Possible mechanism of amyloidosis by keratin 5 and fibulin-4.** First, keratin 5/14 in basal keratinocytes is degraded by enzymes, and then the degradation fragments move to the dermis. Next, the degradation fragments containing the KT5-6 sequence form amyloid-like fibrils with fibulin-4 on elastic fibers. Further accumulation of the degradation fragments enlarges amyloid-like fibrils and forms amyloid deposits.

Cutaneous amyloidosis is commonly observed in skin diseases, but the mechanism has not been well understood. In this paper, we describe the colocalization of K5 and fibulin-4 in cutaneous amyloid deposits by immunohistochemical staining. We localized an active site on K5 for amyloid formation using synthetic peptides that were screened for amyloid-like fibril formation and fibulin-4 binding. Fibulin-4 enhanced KT5-6 amyloid formation. The KT5-6 peptides specifically accumulated in the cutaneous amyloid deposits. These results suggest that K5 degradation fragments containing the KT5-6 sequence form amyloid fibrils with fibulin-4. Previously, fibulin-4 was shown to bind to elastin (Sasaki et al., 2016). On the basis of these findings, we propose a mechanism for cutaneous amyloid formation (Figure 8). First, K5/14 in basal keratinocytes is degraded by proteolytic enzymes, and these fragments move to the dermis. Degradation fragments containing the KT5-6 sequence possess a β-sheet structure and form amyloid-like fibrils with fibulin-4 on elastic fibers. Further accumulation of the degradation fragments enlarges amyloid-like fibrils and forms amyloid deposits.

Previously, K5 was described on the basis of immunohistochemistry as a major component in amyloid deposits, and the amyloid deposits are mainly derived from basal keratinocytes (Chang et al., 2004). Our results also suggest that the components of the amyloid deposits are mainly K5-degradation fragments containing the KT5-6 sequence, which binds to fibulin-4.

Keratin 1 mainly locates in the suprabasal cell layer of the epidermis as keratin 1/10. KT1-6 has a highly homologous sequence to that of KT5-6 and forms amyloid fibrils, but the peptide does not bind to fibulin-4. The difference between the KT5-6 and KT1-6 sequences is only two amino acids, with the Val^13^ and Lys^20^ of KT5-6 replaced with Leu^13^ and Thr^20^ in KT1-6. The Val^13^ and Lys^20^ residues of KT5-6 may be important in fibulin-4 binding.

In this study, we focused on the α-helical rod domain of K5, which assembles into a coiled‒coil structure with the keratin 14 molecule (Lee et al., 2012). KT5-6 locates in the C-terminal end of the α-helical rod domain, but the peptide possessed a β-sheet structure. Previously, a structural switch from α-helical to β-sheet has been suggested to cause diseases owing to unnatural amyloid fibril formation (Armen et al., 2004; Barrow and Zagorski, 1991; Nerelius et al., 2009). It is known that the Aβ42 peptide, which is a partial peptide of the amyloid β protein that causes Alzheimer's disease, has an α-helix structure, and when cleaved, it changes into a β-sheet structure to form amyloid fibrils. The KT5-6 sequence also shows an α-helix structure in the K5/14 molecule, and when cleaved, it changes to a β-sheet structure and forms amyloid fibrils. Furthermore, fibulin-4 promotes amyloid formation in the presence of KT5-6, which is a unique amyloid formation mechanism. It will be important to identify the peptides containing the KT5-6 sequences as a degradation product of K5/14 in the amyloid deposits of patients to confirm this mechanism. Drugs that interfere with the K5/fibrin-4 interaction may be a new target for cutaneous amyloidosis.

In summary, we have shown that KT5-6, a peptide from the α-helical rod domain of K5, formed amyloid fibrils and that the amyloid formation was accelerated by fibulin-4. These results suggest that K5 degradation fragments containing the KT5-6 sequence form amyloid fibrils with fibulin-4 and have the potential to promote cutaneous amyloidosis.

## Materials and Methods

### Patients’ profiles

Patient 1 is a man aged 64 years who visited our hospital because of a tumor in his right lateral malleolus. The tumor was resected and was diagnosed as Bowen’s carcinoma (stage I). Patient 2 is a man aged 53 years who visited our hospital because of a pruritic eruption, and a biopsy specimen was obtained from his left upper arm. The pathological diagnosis was lichen amyloidosis. Patient 3 is a man aged 65 years who visited our clinic because of pruritic papules on his extensor forearms. A biopsy specimen was obtained from the papule, and the pathological diagnosis was lichen amyloidosis. Patient 4 is a man aged 27 years who has had atopic dermatitis and visited our clinic because of a pruritic eruption. A biopsy specimen was obtained from his left forearm. The diagnosis of ripple-pattern lichen amyloidosis concomitant with atopic dermatitis was made. Patient 5 is a female aged 45 years who visited our clinic because of a pruritic eruption. A biopsy specimen revealed direct fast scarlet-positive deposits in the papillary dermis. She was diagnosed as having familial PLCA using whole-exome sequence, which revealed a heterozygous missense mutation in *OSMRβ*, namely c.1891G>T, p.V631L (Ueo et al., 2016).

The research protocol was approved by the medical ethics committee of Nagasaki University (Nagasaki City, Japan) and Oita University (Oita, Japan), and written informed consent was obtained from all patients.

### Antibodies and proteins

Antibodies used in this study were anti-K5 (anti-CK5, number MA5-12596, Invitrogen, Waltham, MA), anti-elastin (15257-1-AP, ProteinTech Group, Rosemont, IL), anti‒fibrillin-1 (AP06122PU-N, Acris Antibodies, Herford, Germany and mab26 [Reinhardt et al., 1996]), anti‒fibulin-4 (D123, Bioworld Technology, St. Louis Park, MN and 1173 [Sasaki et al., 2016]), anti‒fibulin-5 (65018-1-Ig, ProteinTech Group and 1131 [Kobayashi et al., 2007]), anti-versican (2B1, Seikagaku, Tokyo, Japan and 6084 [Murasawa et al., 2018]), anti-LTBP1 (Sc-98275, Santa Cruz Biotechnology, Dallas, TX and Ab39 [Miyazono et al., 1991]), and anti-LTBP4 (Moriya et al., 2011). Anti-LTBP2 was raised in rabbits against the recombinantly prepared protein. Recombinant human fibulin-4, fibulin-5, LTBP1, and LTBP2 were expressed and purified as described previously (Sasaki et al., 2016).

### Immunohistochemistry

Skin specimens obtained from patients 1 and 2 were embedded in paraffin, and those from patients 2‒5 were directly frozen in Tissue-Tek optimal cutting temperature compound (Sakura Finetek, Osaka, Japan). Frozen sections were fixed with cold acetone for 10 minutes before staining.

Antigen retrieval for paraffin-embedded sections was accomplished with 10 mM citrate buffer (pH 6.0) at 95 °C for 10 minutes for anti-K5 and with 0.05% trypsin at 37 °C for 10 minutes for anti-versican and anti-elastin antibodies. Then, sections were incubated with anti-K5 (1:50), anti‒fibrillin-1 (1:50; AP06122PU-N), anti‒fibulin-4 (1: 100; D123), anti‒fibulin-5 (1:100; 65018-1-Ig), anti-versican (1:200; 2B1), anti-LTBP1 (1:100; Sc-98275), anti-LTBP2 (1:100), and anti-LTBP4 (1:100). Frozen sections were stained with anti-K5 (1:50), anti-elastin (1:1,000), mouse monoclonal anti‒fibrillin-1 (50 μg/ml; mab26), affinity-purified anti‒fibulin-4 (5 μg/ml; 1173), anti‒fibulin-5 (1:1,000; 1130), anti-versican (1:100; 6084), anti-LTBP1 (1:1,000; Ab39), anti-LTBP2 (1:1,000), and anti-LTBP4 (1:1,000). The secondary antibodies used were either Alexa 488‒ or Alexa 555‒labeled antimouse antibodies and Alexa 555‒labeled antirabbit IgG. After immunostaining, sections were stained with thioflavin T (Sigma-Aldrich, St. Louis, MO) (Biancalana and Koide, 2010). Normal IgG or preimmune serum, if available, is usually used as negative controls, but they often contain antikeratin antibodies and/or other antibodies that give signals, especially in the skin. Therefore, in this study, the primary antibodies were omitted for negative controls, and those images were acquired with the same exposure time as respective positive images.

### Peptide synthesis

All peptides were manually synthesized by the 9-fluorenylmethoxycarbonyl strategy with a C-terminal amide as previously described (Katagiri et al., 2010). The respective amino acids (Kokusan Chemical, Tokyo, Japan) and biotin (Tokyo Chemical Industry, Tokyo, Japan) were condensed using diisopropylcarbodiimide-*N*-hydroxybenzotriazole on a 4-(2*′*,4*′*-dimethoxyphenyl-Fmoc-aminomethyl)-phenoxy resin (Rink amide resin; Novabiochem, San Diego, CA). The peptides were purified by reverse-phase HPLC on a Mightsil RP-18 GP 250-10 column (Kanto Chemical, Tokyo, Japan) using gradient elution with water/acetonitrile containing 0.1% trifluoroacetic acid. Resulting protected peptide resins were deprotected and cleaved from the resin using trifluoroacetic acid‒thioanisole‒*m*-cresol‒ethanedithiol‒water (80:5:5:5:5, v/v) at room temperature for 3 hours. Crude peptides were precipitated and washed with diethyl ether and then purified by HPLC using a Mightysil RP-18 column (Kanto Chemical) with a gradient of water/acetonitrile containing 0.1% trifluoroacetic acid. The purity and identity of the synthetic peptides were confirmed by HPLC and by electrospray ionization mass spectroscopy. Mass spectroscopy was performed at the Central Analysis Center, Tokyo University of Pharmacy and Life Sciences (Hachioji, Japan).

### CD

Peptides were dissolved at a final peptide concentration of 0.2 mM in 20% (v/v) trifluoroethanol containing 50 mM phosphate buffer (pH 7.0). Spectra were recorded from 260 nm to 185 nm on a J-720 CD spectropolarimeter (JASCO, Tokyo, Japan) in quartz cells with a 2-mm path length at room temperature.

### Electron microscopy

Approximately 5 μl of peptide solutions (1 mM) was placed on parafilm. Then, a carbon-coated 400 mesh copper grid was positioned on the top of the drop for 10 seconds and washed with a droplet of distilled water. The grid was contrasted by adding a drop of 2% uranyl acetate on parafilm and incubating the grid on top of a drop for 10 seconds. Excess liquid was removed gently using absorbing paper. After drying, the grid was viewed in transmission electron microscopy using a HITACHI H-7600 (Hitachi, Tokyo, Japan) at 100 kV. Electron microscopy was performed at the Hanaichi Ultrastructure Research Institute (Okazaki, Japan).

### ELISA

Various amounts of fibulin-4 and fibulin-5 were coated on plastic plates (Thermo Fisher Scientific, Waltham, MA) in PBS at 4 °C overnight. The plates were blocked with 10% nonfat milk in PBS at 37 °C for 1 hour. Biotin peptides (1 nmol/100 μl) were added and incubated at room temperature for 1 hour. After washing with 0.05% Tween 20 in Tris-buffered saline, streptavidin horseradish peroxidase was added and incubated at room temperature for 1 hour. After washing with 0.05% Tween 20 in Tris-buffered saline, SureBlue (3,3*′*,5,5*′*-tetramethylbenzidine peroxidase substrate, KPL, Gaithersburg, MD) was added and incubated at room temperature for 30 minutes. The absorbance was measured at 650 nm.

### Kinetic analysis of KT5-6 amyloid formation with fibulins

A total of 1 μg of fibulin-4, fibulin-5, and BSA was coated on the plastic plate in PBS at 4 °C overnight. The plate was blocked with 10% nonfat milk in PBS at 37 °C for 1 hour. The KT5-6 peptide (5 nmol/100 μl) was added and incubated at room temperature for 1 hour. After washing with 0.05% Tween in Tris-buffered saline, 100 μl of thioflavin T solution (10 μM thioflavin T in 5% hexafluoroisopropanol, 0.1 mM glycine‒sodium hydroxide buffer [pH 8.5]) in the presence of 5 nmol KT5-6 was added. The fluorescent intensities were measured immediately until 720 minutes at an excitation wavelength of 455 nm and emission wavelength at 490 nm.

### Data availability statement

No datasets were generated or analyzed during this study.

## ORCIDs

Fumihiko Katagiri: http://orcid.org/0000-0001-5287-8462

Daisuke Ueo: http://orcid.org/0000-0001-7074-9058

Yumi Okubo-Gunge: http://orcid.org/0000-0001-9607-575X

Aya Usui: http://orcid.org/0000-0003-3591-3099

Sayaka Kuwatsuka: http://orcid.org/0000-0003-4352-4640

Yoshiko Mine: http://orcid.org/0000-0002-7903-0805

Keisuke Hamada: http://orcid.org/0000-0002-7774-0790

Sakuhei Fujiwara: http://orcid.org/0000-0002-3281-0484

Takako Sasaki: http://orcid.org/0000-0003-0514-9931

Motoyoshi Nomizu: http://orcid.org/0000-0002-2264-2907

## Author Contributions

Conceptualization: AUt, MN, TS; Formal Analysis: AUt, MN, TS; Funding Acquisition: MN, TS, AUt; Investigation: FK, DU, YOG, AUs, SK, YM, KH, TS, AUt; Methodology: FK, DU, YOG, AUs, SK, YM, KH, TS, AUt; Project Administration: MN, TS, AUt; Validation: FK, DU, SF, MN, TS; Writing - Review and Editing: MN, TS, YOG, SF
